# Social Networks and Dying at Home: Analysis of Outcomes From 13 Countries Using SHARE

**DOI:** 10.1093/geroni/igaa057.2038

**Published:** 2020-12-16

**Authors:** Stipica Mudrazija, Katherine Ornstein

**Affiliations:** 1 Urban Institute, Washington, District of Columbia, United States; 2 Icahn School of Medicine at Mount Sinai, New York, New York, United States

## Abstract

Social networks are critical for end-of-life decision-making and hands on support and may also impact end-of-life outcomes including location of death. Yet we fail to consider these factors in the context of cultural values and variation in healthcare system financing and resources, e.g., availability of palliative care. Using SHARE, we examined the following factors for 6,391 decedents across 13 countries interviewed in the last year of life: family size, living alone, and size and characteristics of social networks. We compared these characteristics cross-nationally for persons dying at home as opposed to in hospital and other formal settings. While individuals with larger social networks are more likely to die at home, we find a cross-national gradient of higher unmet healthcare needs resulting in overall more home-based deaths. Our findings suggest that individual factors such as family availability must be considered in the context of country-level factors when examining quality end-of-life indicators.

